# An Assessment of Waveform Processing for a Single-Beam Bathymetric LiDAR System (SBLS-1)

**DOI:** 10.3390/s22197681

**Published:** 2022-10-10

**Authors:** Yifu Chen, Yuan Le, Lin Wu, Shuai Li, Lizhe Wang

**Affiliations:** 1 State Key Laboratory of Geo-Information Engineering, 1 Yanta Road, Xi’an 710054, China; 2 School of Computer Science, China University of Geosciences (Wuhan), 388 Lumo Road, Wuhan 430074, China; 3 Donghai Laboratory, Zhoushan 316021, China; 4 School of Geography and Information Engineering, China University of Geosciences (Wuhan), 388 Lumo Road, Wuhan 430074, China

**Keywords:** single-beam, LiDAR, waveform, decomposition bathymetry

## Abstract

The single-beam bathymetric light detection and ranging (LiDAR) system 1 (SBLS-1), which is equipped with a 532-nm-band laser projector and two concentric-circle receivers for shallow- and deep-water echo signals, is a lightweight and convenient prototype instrument with low energy consumption. In this study, a novel LiDAR bathymetric method is utilized to achieve single-beam and dual-channel bathymetric characteristics, and an adaptive extraction method is proposed based on the cumulative standard deviation of the peak and trough, which is mainly used to extract the signal segment and eliminate system and random noise. To adapt the dual-channel bathymetric mechanism, an automatic channel-selection method was used at various water depths. A minimum half-wavelength Gaussian iterative decomposition is proposed to improve the detection accuracy of the surface- and bottom-water waveform components and ensure bathymetric accuracy and reliability. Based on a comparison between the experimental results and in situ data, it was found that the SBLS-1 obtained a bathymetric accuracy and RMSE of 0.27 m and 0.23 m at the Weifang and Qingdao test fields. This indicates that the SBLS-1 was bathymetrically capable of acquiring a reliable, high-efficiency waveform dataset. Hence, the novel LiDAR bathymetric method can effectively achieve high-accuracy near-shore bathymetry.

## 1. Introduction

The bathymetric system of light detection and ranging (LiDAR) is an efficient method for conducting near-shore bathymetry and underwater topographic mapping [1,2,3]. Specifically, bottom- and surface-water signals are usually detected by airborne LiDAR bathymetry (ALB) systems (more commonly known as full-waveform bathymetric LiDAR systems) using the two bands (532 and 1064 nm) that can detect these signals, respectively [4,5]. Dual-frequency ALB systems such as coastal zone mapping and imaging LiDAR (CZMIL) [6], laser airborne depth sounder (LADS) [7,8], Hawkeye II, and Hawkeye III [9] are large and heavy and have strong energy-emission requirements. Hence, their applications are often limited, and single-beam ALB systems that are inexpensive, lightweight, flexible, and have high accuracy and multi-platform adaptability (e.g., unmanned aerial vehicle (UAV) platform) are more preferred in bathymetry and underwater mapping [10].

The in-water transmission of the laser generated by the ALB system is extremely complex and is affected by the reflection of the surface and bottom, the refraction of the air–water interface, and the scattering and absorption of the water column [11,12,13,14]. Consequently, this also generates a complex superposition of echo signal waveforms of different backscattering signals and systematic and environmental noises [15]. To identify echo types and extract the temporal positions of these echo signals, full-waveform data processing methods are primarily applied [16,17,18,19]. These methods are categorized into three types: peak detection, deconvolution, and mathematical simulation. In the peak detection method, the waveform shape of the echo signal is used to identify the waveform peak position, as it indicates energy mutation and reflection, including the maximum peak (MP), zero-crossing, and averaged square difference function (ASDF) [12,20,21,22,23]. Meanwhile, deconvolution is the reverse of the convolution process, in which the received echo signal is considered as the superposed sum of the convolutional responses to scattering, reflection, refraction, absorption, and noise during laser beam propagation [22,24,25,26,27]. In the mathematical simulation method, the underwater transmission process of the laser is simulated [28,29,30]. Previous studies have demonstrated that laser pulses undergo Gaussian distribution, in which surface and bottom reflections, including water column backscatter, can be described and fitted by different Gaussian components [29,31,32,33,34]. However, to achieve strong anti-noise capabilities with high bathymetric accuracy and without additional water parameters, the Gaussian decomposition method can be used, as it has been widely applied in the full-waveform LiDAR bathymetry. Normally, it is combined with various optimization methods such as the nonlinear least squares, expectation–maximization, and Levenberg–Marquardt algorithms [35,36,37,38].

During the process of Gaussian decomposition, several factors influence the final bathymetric accuracy: (1) The overlaps of the sea-surface and -bottom signals in extremely shallow water [39,40]. (2) The signal components, which are difficult to precisely extract owing to both the instrument-derived and random noises from different atmospheric and water environments; for example, the bottom signal would overlap with the noise in relatively deep water, which makes recognition difficult [41]. (3) No universal framework can be effectively applied to all ALB systems, as the Gaussian decomposition of full waveform data is a multi-step and complicated procedure, in which different processes lead to different bathymetric results [42,43].

To overcome the limitations of existing ALB systems applied in nearshore bathymetry, this study designed a low-cost, lightweight, and multi-platform mountable ALB system, single-beam bathymetric LiDAR system 1 (SBLS-1). However, determining its bathymetric capacity and accuracy is very important, as it is necessary to facilitate the development and improvement of the system for future applications in nearshore assessments. As the traditional Gaussian decomposition method has significant limitations, a novel data processing procedure was developed for SBLS-1, in which an automatic channel-selection method was employed based on water depth to improve the bathymetric results of the extremely shallow water. An adaptive extraction method was also adopted based on the cumulative standard deviation of the peak and trough to extract the signal segment and eliminate system and random noise. With this, a minimum half-wavelength Gaussian iterative decomposition (MHGID) is proposed to improve the detection accuracy of the surface- and bottom-water waveform components and ensure bathymetric accuracy and reliability. This study aims to develop a technical procedure to process the full-waveform data of SBLS-1 and verify its bathymetric accuracy using two study areas. This study can potentially serve as a basis for future technical improvements of SBLS-1 and other ALB systems, thus further broadening its application in coastal areas.

## 2. Methods

In full-waveform LiDAR bathymetry, laser echo signals are regarded as the convolution of the laser pulse and various objects during air and water transmission [44,45,46,47], in which systematic and environmental noises contribute to the echo signals. Previous research has shown that more than 98% of an echo signal can be fitted using a series of Gaussian curves [44]. The echo waveform was decomposed using a Gaussian waveform to acquire several waveform components. The surface- and bottom-water components were then detected and extracted from several waveform components. Subsequently, their temporal positions were obtained to determine the time interval (∆*t*) and calculate the near-shore bathymetry using Equation (1). The water refractive index of the laser is represented by $n_{w}$, which is 1.34. The *θ* and $c_{0}$ denote the laser incident angle and speed of light.

(1) $$D=\frac{c_{0}\cdot \Delta t\cdot \cos[\arcsin(\frac{\sin\theta }{n_{w}})]}{2n_{w}}$$

**Figure 1:**
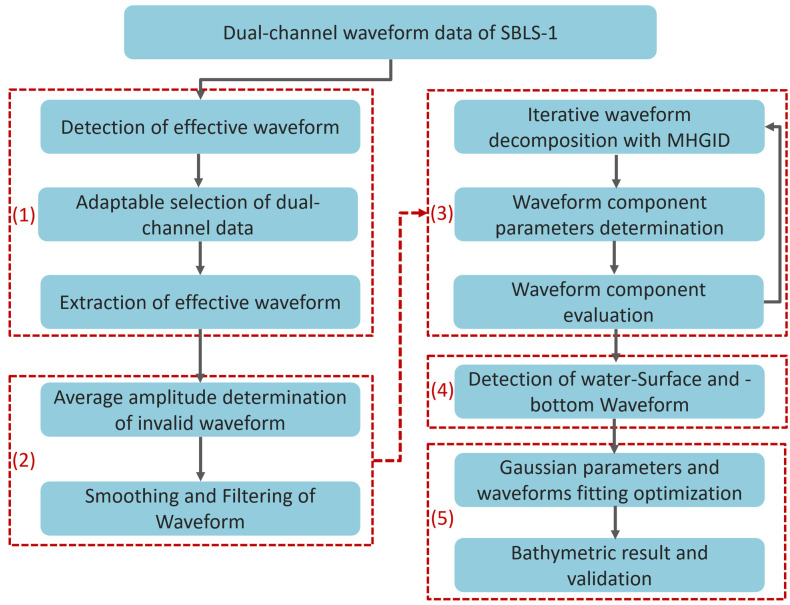
Flow chart of the bathymetric method for SBLS-1.

The accuracy and reliability of bathymetry were improved by adopting a single-beam laser pulse with dual-channel measurements, including both shallow and deep-water channels. To obtain these desired characteristics, a novel LiDAR bathymetric method was used, which involved five phases: (1) signal segment detection and data channel selection, (2) waveform denoising and smoothing, (3) iterative waveform decomposition with a minimum half-wavelength Gaussian iterative decomposition (MHGID), (4) detection of the surface- and bottom-water waveforms, and (5) Gaussian parameters and waveform fitting optimization. A flowchart of the proposed method is shown in Figure 1.

### 2.1. Signal Segment Detection and Data Channel Selection

**Figure 2:**
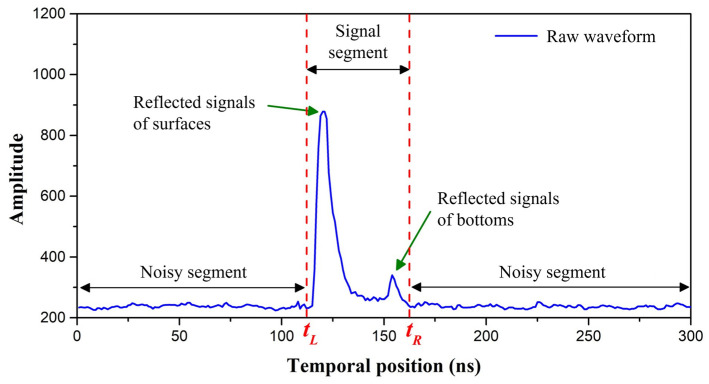
Diagram of adaptive waveform extraction from the raw waveform.

To improve the calculation efficiency and bathymetric accuracy, the identification and extraction of the signal segment were necessary, as they reduced systematic and environmental noise. Here, the signal segment represents the segment containing the characteristic optical response of the target (sea surface and bottom). Figure 2 shows the signal and noisy segment of the raw waveform.

An adaptive extraction method based on the cumulative standard deviation of the peak was used. Here, the time series of the maximum and minimum local amplitudes were obtained. The positive-direction local maximum sequence was represented by a time response amplitude function, $f\left(t_{i}^{u}\right)$, in which $t_{i}^{u}$ is the temporal position sequence and *i* represents the number of local maxima. The positive-direction local minimum sequence is described by $f\left(t_{j}^{d}\right)$, where $t_{j}^{d}$ and *j* are the temporal position sequence and number of local minima. Correspondingly, the cumulative standard deviation sequences of the positive-direction maximum and minimum sequences, $\sigma_{u}^{L}\left(i\right)$ and $\sigma_{d}^{L}\left(j\right)$, are expressed by Equation (2):

(2) $$\{\begin{array}{c} \begin{array}{cc} \sigma_{u}^{L}\left(i\right)=\sqrt{\frac{\sum_{i=1}^{N_{num}^{L}}{\left[f\left(t_{i}^{u}\right)-{\bar{f}}_{u}\left(N_{num}^{L}\right)\right]}^{2}}{N_{num}^{L}}} & \left(i=1,\cdots ,n\right) \end{array}\\ \begin{array}{cc} \sigma_{d}^{L}\left(j\right)=\sqrt{\frac{\sum_{j=1}^{M_{num}^{L}}{\left[f\left(t_{j}^{d}\right)-{\bar{f}}_{d}\left(M_{num}^{L}\right)\right]}^{2}}{M_{num}^{L}}} & \left(j=1,\cdots ,m\right) \end{array} \end{array}$$

where *L* denotes the positive direction. The average values of the local maximum and minimum sequences are represented by ${\bar{f}}_{u}\left(N_{num}^{L}\right)$ and ${\bar{f}}_{d}\left(M_{num}^{L}\right)$, which are calculated using Equation (3).

(3) $$\{\begin{array}{c} {\bar{f}}_{u}\left(N_{num}^{L}\right)=\frac{1}{N_{num}^{L}}\sum_{i=1}^{N_{num}^{L}}f\left(t_{i}^{u}\right)\\ {\bar{f}}_{d}\left(M_{num}^{L}\right)=\frac{1}{M_{num}^{L}}\overset{M_{num}^{L}}{\underset{j=1}{\sum }}f\left(t_{j}^{d}\right) \end{array}$$

Considering that the current value of the cumulative standard deviation, $\sigma_{u}^{L}$, is $k_{1}$-times greater than the value of the cumulative standard deviation in the previous calculation, the left temporal position, $t_{L}$, of the signal segment can be represented by Equation (4).

(4) $$\begin{array}{cc} t_{L}=t_{j}^{d}, & \left[\begin{array}{cc} \sigma_{u}^{L}\left(N_{num}^{L}\right)\geq k_{1}\sigma_{u}^{L}\left(N_{num}^{L}-1\right), & t_{N_{num}^{u}-1}^{u}<t_{j}^{d}<t_{N_{num}^{u}}^{u} \end{array}\right] \end{array}$$

Similarly, the cumulative standard deviation sequences of the negative-direction maximum and minimum sequences are represented as $\sigma_{u}^{R}\left(p\right)$ and $\sigma_{d}^{R}\left(q\right)$. Owing to the gradual decrease in the reflected energy from the bottom water, the temporal position of the signal segment on the right side, $t_{R}$, was different from that on the left side; hence, this can be identified as the mean values of $\sigma_{u}^{L}$ and $\sigma_{d}^{L}$ in the invalid waveform. In Equation (5), the mean value is represented by $\sigma_{mean}^{L}$. The value of $k_{2}$ was 1.5 times greater than the average amplitude of the invalid waveform [9].

(5) $$\begin{array}{cc} t_{R}=t_{q}^{d}, & \begin{array}{cc} {}[\frac{\sigma_{u}^{R}\left(p\right)+\sigma_{u}^{R}\left(q\right)}{2}\geq k_{2}\sigma_{mean}^{L}, & t_{P_{num}^{R}}^{u}<t_{q}^{d}<t_{P_{num}^{R}+1}^{u}] \end{array} \end{array}$$

A segmented field-of-view receiver is designed for SBLS-1 to improve the bathymetric accuracy in shallow water and the maximum detection depth in deep water. Consequently, a large annular FOV is adopted to acquire the deep-water signals (deep-water channel), and a center FOV is used to acquire the shallow-water signals (shallow-water channel) [39]. Hence, a challenge in processing the multi-channel data of SBLS-1 is to select the appropriate channel (shallow-water or deep-water channel) in water with different depths. Notably, the standard deviations of the two-channel data differed in the signal segment, in which the amplitude for the shallow-water channel had a standard deviation ($\sigma_{y\left(t\right)}^{S}$) that was greater than that of the deep-water channel in shallow waters ($\sigma_{y\left(t\right)}^{D}$). Meanwhile, an inverse relationship was observed in deep waters. To adapt to these conditions, the channel selection was conducted using Equation (6), in which $k_{3}$ is the judgment parameter and $S_{data}$ and $D_{data}$ represent the selected waveform data from the shallow or deep-water channels as the input data for the subsequent process.

(6) $$\{\begin{array}{c} S_{data}=\sigma_{y\left(t\right)}^{S}\geq k_{3}\sigma_{y\left(t\right)}^{D}\\ D_{data}=\sigma_{y\left(t\right)}^{S}<k_{3}\sigma_{y\left(t\right)}^{D} \end{array}$$

### 2.2. Waveform Denoising and Smoothing

**Figure 3:**
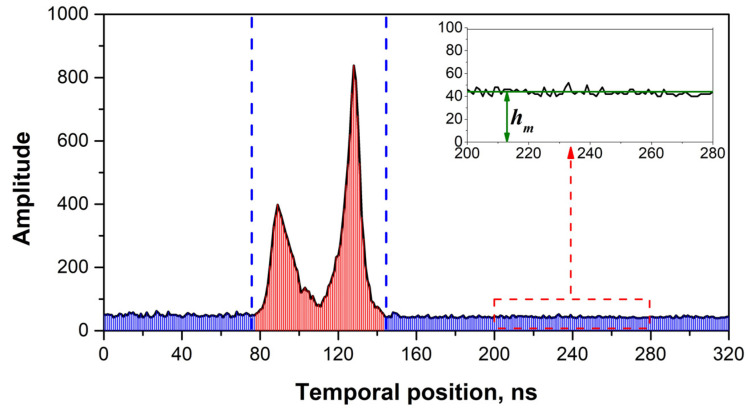
Determination of sigma in the Gaussian filter through the noisy segment. The green line in this figure represents the horizontal line of an invalid waveform after filtering; *h_m_* is the calculated average value of the amplitude of the noisy segment.

Systematic and random noises were still observed following signal segment extraction. Hence, a Gaussian filter, which typically eliminates random noise [41], was applied. However, determining the appropriate sigma value for the Gaussian function was difficult. To address this, different sigma values ranging from 0.1 to 1.5 at intervals of 0.1 were used on the noise of the waveform. The appropriate sigma value was considered as the input value, as the invalid waveform was smoothed to a horizontal line. The average value of the amplitude of the invalid waveform was then calculated to eliminate the systematic noise (Figure 3). In addition, the standard deviation of the raw and filtered signal segments, represented by $\sigma_{\varepsilon }$, was calculated and used as a parameter in the Gaussian decomposition process described in the following section.

### 2.3. Iterative Waveform Decomposition

**Figure 4:**
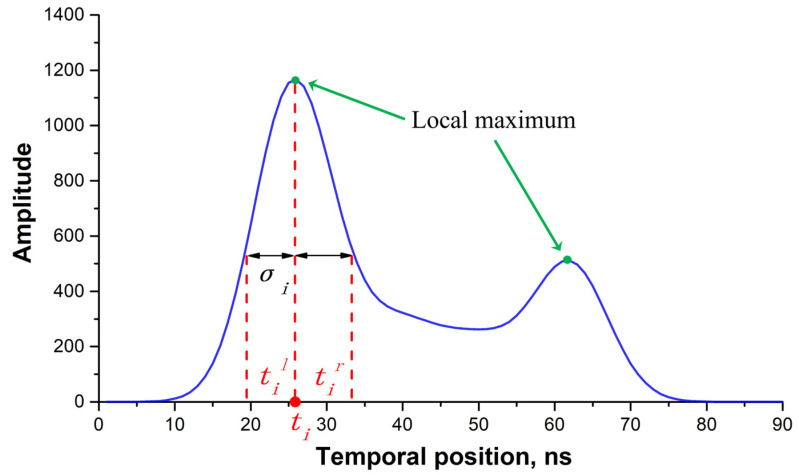
Diagram of the minimum half-wavelength in waveform data. The blue curve represents the signal segment after noise filtering; the red dashed lines denote the temporal position of the peak and half-peak amplitude.

To achieve an iterative waveform decomposition and obtain the initial parameters of each Gaussian component, the MHGID method was proposed and applied. Firstly, the local maximum points of the waveform were detected using the first-order differential, which is represented by green dots in Figure 4. Although the waveforms of the signal segments were smoothed, there were still a lot of local maximum points. To reduce the number of inaccurate Gaussian components, only points with an amplitude of more than three times $\sigma_{\varepsilon }$ were used in the Gaussian waveform decomposition. The multiple was an experience value based on our previous experiments. The corresponding waveform parameters of each Gaussian component are described by the amplitude $a_{i}$, temporal position $t_{i}$, and waveform width $\sigma_{i}$, as formulated in Equation (7).

(7) $$G\left(t\right)=\overset{n}{\underset{i=1}{\sum }}a_{i}\exp\left[\frac{-{\left(t-t_{i}\right)}^{2}}{2\sigma_{i}^{2}}\right]$$

The minimum half-wavelength Gaussian function was used to acquire the waveform width, $\sigma_{i}$. For each peak point, half of the peak amplitude intersects the waveform, generating two cross points, $t_{i}^{l}$ and $t_{i}^{r}$, on either side of the temporal position ($t_{i}$, Figure 4). Subsequently, the waveform width, $\sigma_{i}$, was calculated using the absolute minimum distance between $t_{i}$ and $t_{i}^{l}$ and $t_{i}^{r}$, as represented in Equation (8).

(8) $$\sigma_{i}=\frac{\min(\left|t_{i}-t_{i}^{l}\right|,\left|t_{i}-t_{i}^{r}\right|)}{\sqrt{2\ln(2)}},i=1,2,\cdots n$$

**Figure 5:**
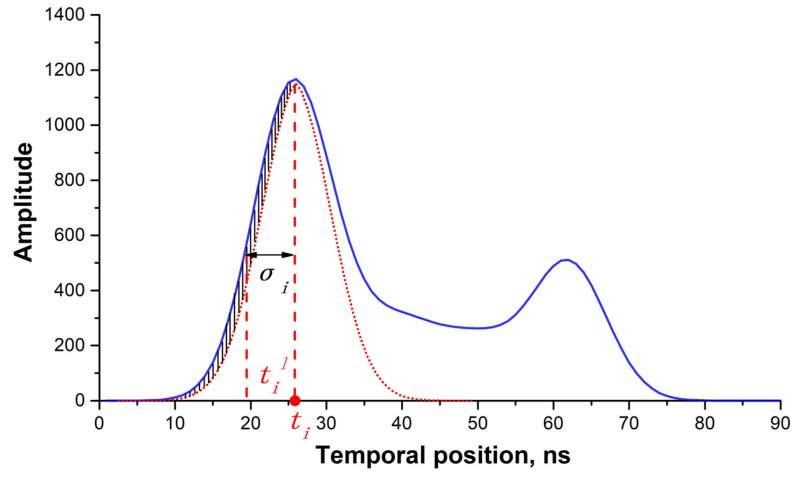
Waveform decomposition and parameter optimization based on the standard deviation of the left half of the waveform. The dashed red curve is the fitted Gaussian curve; the blue curve indicates the signal segment.

To improve the accuracy of $\sigma_{i}$, the value was further refined using the standard deviation between the Gaussian line and the signal segment of the left half of the waveform (Figure 5). The changing step of $\sigma_{i}$ for the Gaussian line was set as 0.1, with a range of −2 to 2. As the standard deviation reached the minimum, the corresponding refined waveform width $\sigma_{i\_refine}$ was then determined.

### 2.4. Detection of Surface and Bottom Water Waveforms

The attenuation of the laser transmission process resulted in the widening of the echo waveform, accompanied by a decreasing intensity. Generally, the amount of attenuation is determined by the type of medium, propagation distance, and other environmental factors. According to previous research, the interval between adjacent components should be greater than the width of one laser pulse, along with the presence of one clear waveform peak [48]; otherwise, the temporal positions of different components may be difficult to distinguish. In this study, the interval constraint condition for the adjacent Gaussian component should meet Equation (9), as follows:

(9) $$\left(t_{i+1}-t_{i}\right)>\sigma_{sys}$$

where $\sigma_{sys}$ represents the half-width of the system laser pulse of SBLS-1. The center of the Gaussian waveform components is represented by $t_{i}$ and that of the next waveform is denoted by $t_{i+1}$. The waveform components contradicted by Equation (9) should then be combined with adjacent components to obtain a new waveform component. Therefore, all the Gaussian waveform components were processed until each satisfied Equation (9).

**Figure 6:**
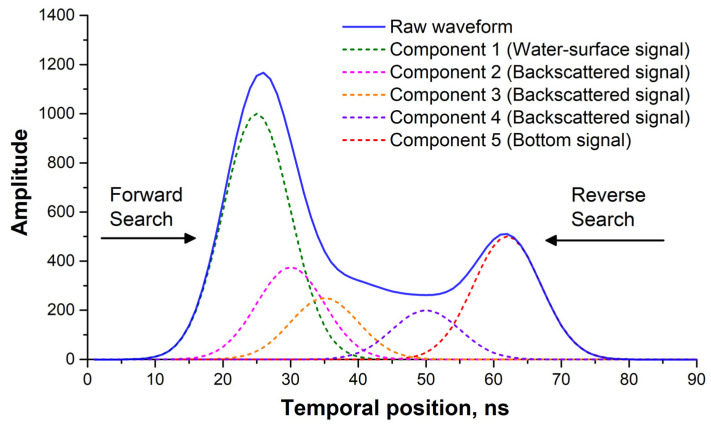
Waveform decomposition and detection of surface- and bottom-water waveform components.

The detection and determination of the surface- and bottom-water waveform components were crucial for bathymetric accuracy [13,22]. Hence, the influence of water-body backscattering should be considered, in addition to the separation of the waveform components that describe water backscattering and the surface and bottom waters. The mean amplitude of the invalid waveform, which was separated from the valid waveform in Section 2.1, was counted. It was then set as the threshold noise value and the maximum amplitude of each Gaussian waveform component in the time series, counterclockwise, was subsequently compared to the noise. As the maximum amplitude of the first waveform component became greater than the threshold value, the waveform component was determined as the surface-water echo. Along the time series, the bottom-water echo was detected as the first waveform component whose maximum amplitude was greater than the threshold value of noise. Figure 6 shows a detection diagram of the surface- and bottom-water waveform echoes.

### 2.5. Gaussian Parameters and Waveform Fitting Optimization

To ensure bathymetric accuracy with minimal fitting errors between the fitted waveform components and the signal segment, the nonlinear least squares Levenberg–Marquardt (LM) algorithm was used to optimize the parameters of the Gaussian components [33], as formulated in Equation (10), and the error equation of all components was obtained using Equation (11). The formulas of the iterative optimization and iterative termination conditions are given based on the LM theory [35,39].

(10) $$G_{i}\left(t,a_{i},t_{i},\sigma_{i\_refine}\right)=a_{i}\exp\left[\frac{-{\left(t-t_{i}\right)}^{2}}{2\sigma_{i\_refine}^{2}}\right],\left(i=1,2,\cdots n\right)$$

(11) $$F\left(t,p\right)=G\left(t,p\right)-\overset{n}{\underset{i=0}{\sum }}G_{i}\left(t,a_{i},t_{i},\sigma_{i\_refine}\right)=0,p∼\left(a_{i},t_{i},\sigma_{i\_refine}\right)$$

## 3. Materials

### 3.1. Single-Beam Bathymetric LiDAR System

**Figure 7:**
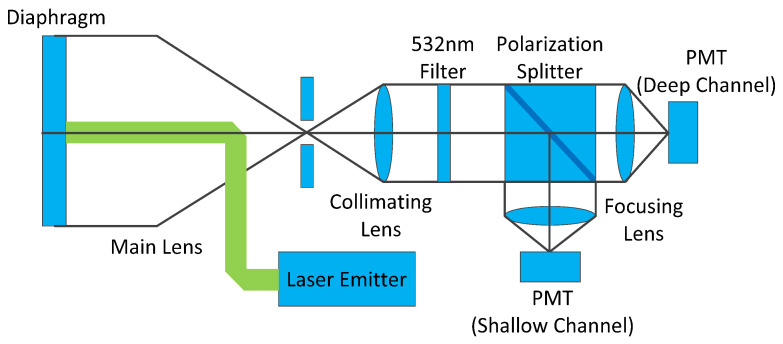
Diagram of the optical path structure of SBLS-1.

SBLS-1 is a single-beam bathymetric LiDAR scanner equipped with a 532 nm laser emitter. The designed bathymetric capacity for SBLS-1 ranges from 0.2 to 20 m in transparent water. The repetition frequency was 100 Hz, and the pulse width was 10 ns. The major payload parameters of SBLS-1 are summarized in Table 1. A coaxial optical path strategy was adopted for SBLS-1 to ensure a compact and efficient optomechanical structure. A segmented field-of-view receiver is designed for SBLS-1 to improve the bathymetric accuracy in shallow water and the maximum detection depth in deep water. The center and annular receivers were adopted to acquire shallow and deep-water echo signals in two data channels. The shallow-water echo receiver had a small reception FOV, while the deep-water echo receivers had a large reception FOV. Figure 7 shows the optical structure of the prototype system used in SBLS-1.

### 3.2. Study Area and Data Acquisition

**Figure 8:**
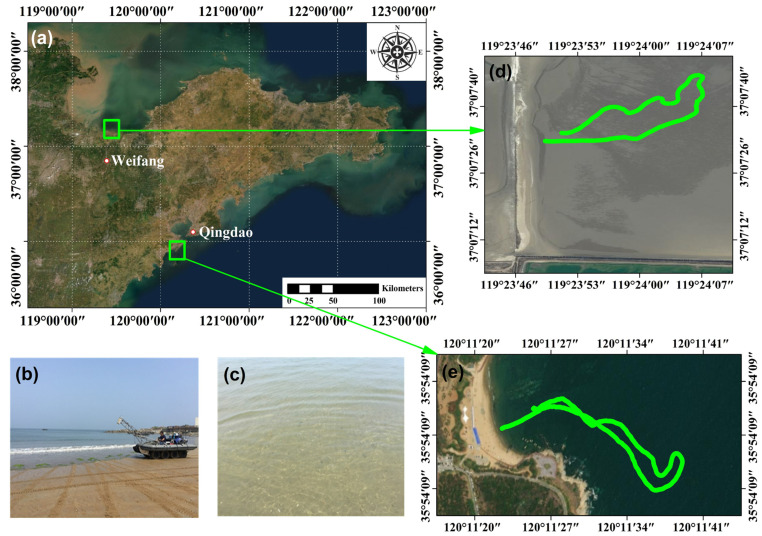
(**a**) Study area locations (green boxes). (**b**) Image of the equipment platform. (**c**) Representative image of the water environment in the study areas. (**d**,**e**) Measurement trajectories represented by green lines at Qingdao (**d**) and Weifang (**e**).

The validation and assessment of SBLS-1 involved the selection of two test fields located in Qingdao and Weifang in Shandong Province (Figure 8a). The bathymetric LiDAR system was installed onto a modified eight-wheel amphicar to obtain the curvilinear trajectory data from shallow to deep waters (Figure 8b). The water properties of the two areas were observed to be similar, with a Secchi disk transparency of 1.9 m for Weifang and 2.2 m for Qingdao, as shown in Figure 8c. Data in Weifang were acquired on 24 December 2018, from 15:43 to 16:10, whereas the data in Qingdao were acquired on 27 June 2019, from 15:01 to 15:26. The trajectories of the data in Weifang and Qingdao are illustrated in Figure 8d,e, in which it showed extreme irregularities. As GPS data were obtained simultaneously during measurement acquisition, these were corrected using the relative position between the GPS antenna center and SBLS-1. However, the real-time coordinate information acquired by the GPS system was not downlinked to the control system of the eight-wheel amphicar. Hence, the survey route was determined as a simple “go and return” route near the coastline to avoid loss during the long-term measurements on the sea surface and ensure personnel safety. Additionally, the in situ bathymetry data that were obtained using the HD-max single-beam echo system developed by Hi-Target Co. Ltd. was equipped on the amphicar. The maximum detection capacity of the HD-max was at a depth of 300 m, with a nominal accuracy of ± 1 cm + 0.1% × depth [49]. The HD-max was installed on the back of the modified eight-wheel amphicar to continuously acquire the in situ water depths while the SBLS-1 was working. Finally, in situ water depths were used to validate and evaluate the bathymetric capability of SBLS-1, including the accuracy of the proposed method.

## 4. Results and Discussion

### 4.1. Signal Segment Extraction and Data Channel Selection

**Figure 9:**
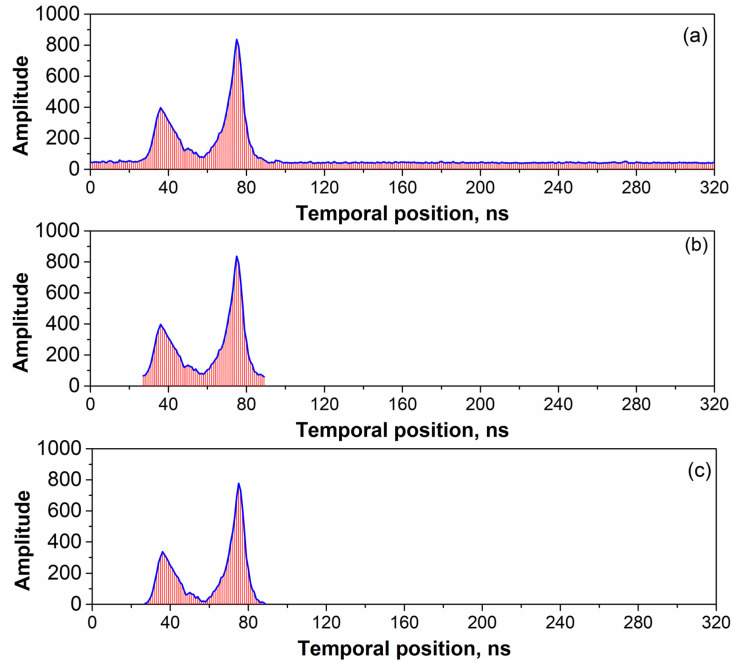
Extraction of the signal segment. (**a**) Raw echo waveform. (**b**) Adaptive extraction of the signal segment. (**c**) Signal segment after noise elimination.

In conventional approaches, all waveform data are used directly for each echo pulse. Meanwhile, in some approaches, threshold values are used to extract the signal segment; however, this method may not be adaptable to various waveform data [23,25]. In this study, the proposed adaptive extraction method was determined to be capable of overcoming this issue by automatically and precisely selecting the signal segment, as indicated by the cumulative standard deviation of the peak and trough. The raw waveform is shown in Figure 9a, while the signal segment extracted using the proposed adaptive method is shown in Figure 9b. In the latter, the noise-derived amplitude was clearly observed along the bottom of the signal segment; therefore, the average amplitude of the remaining waveform can be calculated and was used to eliminate system and random noise, as shown in Figure 9c.

**Figure 10:**
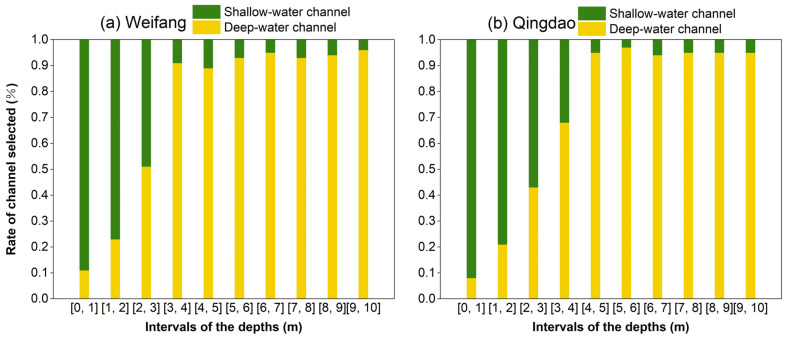
Channel selection ratios at various water depths for the test fields at (**a**) Weifang and (**b**) Qingdao.

Figure 10 shows the selection rates of the shallow and deep-water channels at different water depths ranging from 0 to 10 m at the two test fields. For the test field at Weifang, data from the shallow-water channel were used for depths ranging from 0 to 2 m, while data from the deep-water channel were used for depths >2 m (Figure 10a). At the Qingdao test field, shallow-water channel data were used for depths ranging from 0 to 3 m, while deep-water channel data were used for depths >3 m (Figure 10b). Figure 10 illustrates that different water environments require different channel selections, resulting in different echo waveforms. However, the overlap between the water-surface and -bottom signals was inevitable owing to the limitations of the laser pulse duration and receiver bandwidth [39]. Moreover, the energy of the bottom signals decreased with increasing water depth, leading to the failure of the signal extraction. Therefore, the design of multi-channel and multi-FOV can be extremely important for bathymetric assessments using ALB systems in various water environments. This includes the selection of appropriate channels to obtain high accuracy in near-shore bathymetry [10,41]. Although the method proposed in this study could automatically select an appropriate channel, its performance in other types of water requires further validation.

### 4.2. Waveform Decomposition Results

**Figure 11:**
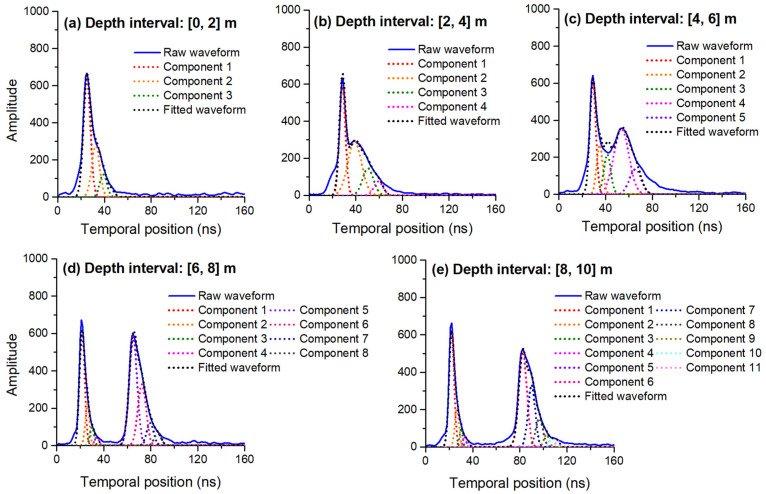
Waveform decomposition diagrams showing waveform components identified using MHGID. The different waveform components are represented by colored dashed lines. The fitted curve reconstructed through the decomposition waveform components is represented by a black dashed line.

Based on the previously extracted signal segment, the waveform decomposition was performed using the MHGID method. Figure 11 shows the results of the waveform decomposition for different water depths in the Weifang test field. It was observed that the surface- and bottom-water echo waveforms became significantly more distinguishable with increasing water depth. In shallow areas ranging from 0 to 4 m, the interval between the surface- and bottom-water echo waveforms was small, causing the superposition of their waveforms, which prevented the distinction between their wave peaks. In areas with a depth of >4 m, the waveforms exhibited fewer overlaps and the surface- and bottom-water wave peaks became easily distinguishable. Here, the waveform superposition gradually decreased with increasing water depth until its eventual complete separation.

The numbers of waveform components at different water depths are presented in Table 2. Considering the entire range of depths of 0–10 m, the average number of components observed at the Weifang test field was greater than that of the Qingdao area. At a depth of 2 m, an average of 2.81 and 2.47 waveform components at the Weifang and Qingdao test fields were observed. Meanwhile, 4.46 and 3.82 components were observed for depths from 2 to 4 m. Similarly, the number of waveform components increased with increasing water depth, reaching 11.31 and 10.56 for depths from 8 to 10 m. Except for the 4–6 m depth interval, the average number of waveform components observed at each water depth interval was greater at the Weifang test field than that of the Qingdao test field, indicating that the backscatter of the water column at Weifang was greater. However, the bathymetric accuracy of MHGID needs to be further validated using experiments in areas where the water is relatively turbid, such as inland lakes.

To validate the accuracy of the MHGID method, the average value and standard deviation of the amplitude between the fitted waveform and signal segment were calculated (Table 3). For the test fields at Weifang and Qingdao, the average amplitude values of the entire water depth range were 9.65 ± 15.56 and 9.57 ± 15.28, and similar results were obtained for each water depth interval. In general, the decomposition results of MHGID were consistent with the raw waveform of SBLS-1, with the difference in amplitude being less than 2%.

### 4.3. Optimization of Waveform Parameters

To analyze and validate the optimization results in detail, changes in the surface- and bottom-water waveform parameters before and after the LM optimization were calculated at the Weifang test field at the 0–10 m water depth (Table 4). $\Delta A_{S}$,$\;\Delta \mu_{S},$ and $\Delta \sigma_{S}$ represent the change in amplitude, temporal position of the waveform peak, and waveform width of the surface-water component, respectively. $\Delta A_{B}$,$\;\Delta \mu_{B},$ and $\Delta \sigma_{B}$ denote changes in the corresponding parameters of the bottom-water component. As shown in Table 4, the temporal position of the surface-water waveform component was displaced clockwise following LM optimization, which resulted in a reduction in the waveform width. In contrast, the temporal position of the bottom-water waveform component was displaced to the right at the 4–6 m water depth but was displaced to the left at other depths. Here, the waveform width of the surface-water component decreased following LM optimization, whereas it increased for the bottom-water component. Furthermore, a slight decrease in amplitude was observed for both the surface- and bottom-water waveform components.

From the data provided in Figure 11 and Table 4, the leftward displacement of the surface-water waveform component could be primarily attributed to the water column backscatter, whereas the rightward displacement of the bottom-water waveform component could be attributed to the trailing phenomenon observed in the bottom-water echo waveform. These changes result from interactions between the waveform components of the surface and bottom waters and water backscatter. Hence, an enhanced fit of the raw waveform, with minimal fitting error, can be indicated by the generation of the LM optimization changes of the wave width and the amplitude of surface- and bottom-water waveform components.

### 4.4. Bathymetric Results and Accuracy Assessment

**Figure 12:**
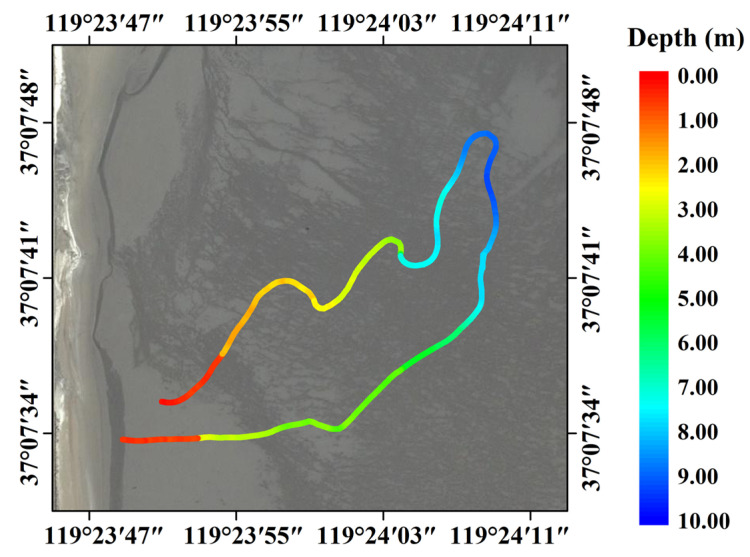
Distribution trajectory and bathymetric results at the Weifang test field.

**Figure 13:**
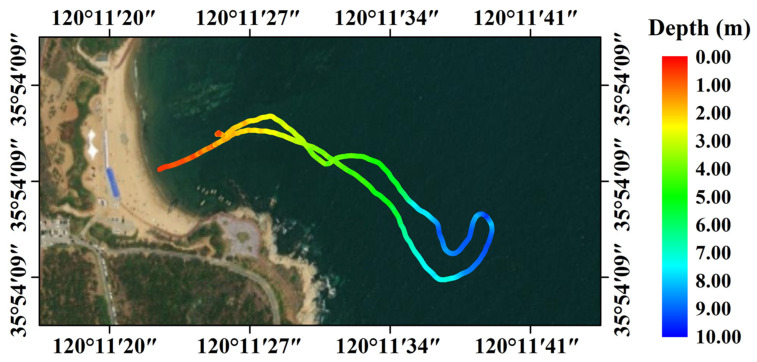
Distribution trajectory and bathymetric results at the Qingdao test field.

In this study, the bathymetric results obtained using SBLS-1 were compared to in situ data to validate the accuracy of the proposed bathymetric method. Figure 12 and Figure 13 show the measurement trajectories and bathymetric results acquired at the Qingdao and Weifang test fields. The modified amphicar shown in Figure 8b was run on a looping trajectory from the shoal of the coastline to the deep-water region. The acquired bathymetry changes, from shallow water to a maximum depth of approximately 10 m and vice versa, were visually consistent with actual water depth changes at both test fields. Owing to the limitations in the measured trajectories and underwater topography, the obtained trajectories exhibited irregular curves that were not uniformly distributed across the entire study area.

The trajectory length, echo waveform number, and signal segment detection ratio, including the laser point density, were measured at the two test fields (Table 5). Using these evaluation indices, the bathymetric capability and efficiency of SBLS-1 and the proposed method were validated and further assessed. The results indicate that the trajectory length at Qingdao and Weifang was 1781.5 and 3671.4 m, and the acquired number of echo waveforms was 84,579 and 102,454. Here, the detection ratio of the signal segment is indicative of the ratio of detectable waveforms to total waveforms in the entire measurement trajectory (percentage). Using this ratio value, the bathymetric efficiency of the MHGID method and the measurement capability of the SBLS-1 bathymetric system were validated, and the detection ratio of the signal segment was found to have reached 87% and 81% (Table 5).

**Figure 14:**
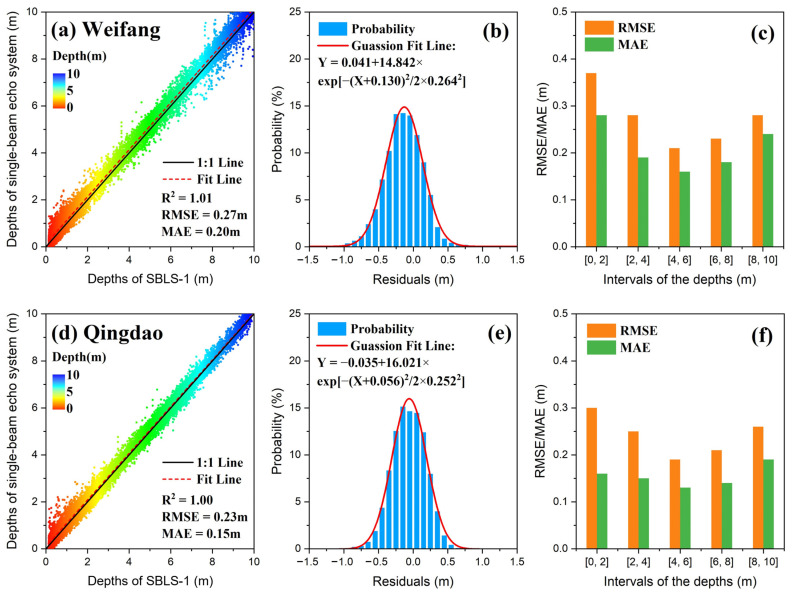
Validation of bathymetric accuracy at the Weifang (**a**–**c**) and Qingdao (**d**–**f**) test fields.

As shown in Figure 14, the bathymetric results obtained using SBLS-1 and MHGID were compared to in situ data. The scatter diagram in Figure 14a demonstrates the relatively high bathymetric accuracies of the MAE and RMSE at the Weifang test field, reaching 0.20 and 0.27 m. The residuals of the bathymetric results between the SBLS-1 and the in situ data were normally distributed, with a deviation of -0.13 m from the true value (Figure 14b). The value of RMSE improved from 0.37 m at a depth interval of 0–2 m to 0.21 m at a depth interval of 4–6 m. However, it decreased to 0.28 m at a depth interval of 8–0 m. Meanwhile, the value of MAE improved from 0.28 m at a depth interval of 0–2 m to 0.16 m at a depth interval of 4–6 m (Figure 14c); however, the accuracy fell to 0.24 m at a depth interval of 8–10 m. Similar trends were observed at the Qingdao test field; however, the bathymetric accuracies of the MAE (0.15 m) and RMSE (0.23 m) were slightly lower than those at the Qingdao test field (Figure 14d). Notably, the residuals of the bathymetric results at Qingdao were unbiased with a normal distribution with a deviation of -0.056m (Figure 14e), and the results indicate that RMSE and MAE values were fluctuating throughout (Figure 14f).

Slightly lower RMSE values were observed at the Qingdao test field compared to those of Weifang (compare Figure 14b,d). This may be due to the presence of higher water transparency in Qingdao. Additionally, the lower RMSE values at shallow depths (0–2 m) may have resulted from the superposition of surface- and bottom-water echoes. As presented in Figure 11, the peaks of surface- and bottom-water waveforms gradually became easily distinguishable in deeper water; hence, an improvement in the bathymetric accuracy with increasing water depth. However, the bathymetric accuracy eventually began to decrease with increasing water depth owing to the decreasing reflected laser energy and increasing water backscatter. As it reached a certain depth, the amplitude of the bottom-water echo became weak and indistinguishable from the noise, indicating that the depth limit had been reached. The maximum depths detected at Weifang and Qingdao were 10.38 m and 9.89 m, which was not within the maximum bathymetric capacity of the SBLS-1. The processing of waveforms in extremely shallow areas and relatively deep areas significantly affects the bathymetric accuracy of the ALB system. Therefore, more extensive work in these areas is necessary to further investigate the bathymetric accuracy and capacity of SBLS-1.

The adaptive extraction method proposed in this study can effectively eliminate noise from the raw waveform echo. The sufficient extraction of the signal segment can also be acquired to ensure the efficiency and accuracy of subsequent waveform processing. In this study, the bathymetric capability and accuracy of SBLS-1 were limited to two areas with waters with optically and inherently similar water characteristics. Based on the comparison between the high-accuracy bathymetric results and reference data, the proposed method was inferred to be applicable in other areas such as inland lakes and distant reefs from the mainland. However, maximum effective depth ranges are still necessary to be determined as they are closely related to the optical properties of water.

## 5. Conclusions

In this study, SBLS-1, a lightweight and low-energy-consuming system was designed for high-accuracy near-shore bathymetry. To achieve this, an adaptive extraction method based on the cumulative standard deviation of the peak and trough was used to extract the signal segment and eliminate system and random noise. To ensure bathymetric accuracy, an automatic channel-selection method was used to adjust the bathymetric mechanism between the dual, shallow-water, and deep-water data channels at each water depth. The MHGID was used to improve the detection accuracy of surface- and bottom-water waveform components and ensure high bathymetric accuracy and reliability. The comparison with the in situ water depth data resulted in RMSE values of 0.23 and 0.27 m at the Qingdao and Weifang test fields. The results demonstrate that SBLS-1 was capable of producing reliable bathymetric data that can be used to generate a high-efficiency waveform dataset. Furthermore, the proposed novel LiDAR bathymetric method was also observed to effectively achieve highly accurate near-shore bathymetry. The use of the SBLS-1 can promote bathymetric methods to obtain high-accuracy measurements of land–sea topographies in different environments. However, more extensive work in areas with optically and inherently different water characteristics is necessary to further determine the bathymetric capacity and accuracy in other coastal conditions and expand its use in future applications. It is recommended that the system hardware and the data processing intelligence of the software are further improved and optimized to continually contribute to ALB system development.

## Figures and Tables

**Table 1:** Payload parameters of SBLS-1.

Payload Parameter	Indicator
Laser wavelength	532 nm
Mission/repetition frequency	100 Hz
Pulse width	10 ns
Pulse energy	5–20 μJ
Beam quality	M2 < 1.3

**Table 2:** Average numbers of waveform components at various water depths for the two test fields.

Test Field	Water Depth Interval (m)	0–2	2–4	4–6	6–8	8–10	All Depths
Weifang	Average of components	2.81	4.46	5.51	7.89	11.31	6.40
Qingdao	Average of components	2.47	3.82	5.69	7.35	10.56	5.98

**Table 3:** Difference in amplitude between the fitted waveform and the signal segment at different depths at the two test fields.

Test Fields	Water Depth Interval (m)	0–2	2–4	4–6	6–8	8–10	Average
Weifang	Mean difference	9.98	9.93	9.61	9.37	8.94	9.57
	Standard deviation	17.35	14.98	15.21	14.55	14.31	15.28
Qingdao	Mean difference	10.06	10.01	9.85	9.25	9.06	9.65
	Standard deviation	17.54	15.16	15.73	14.79	14.58	15.56

**Table 4:** Changes in the surface- and bottom-water waveform parameters before and after LM optimization.

**Depths (m)**	Surface Water Component Parameters			Bottom Water Component Parameters		
	$\Delta A_{S}$	$\Delta \mu_{S}$	$\Delta \sigma_{S}$	$\Delta A_{B}$	$\Delta \mu_{B}$	$\Delta \sigma_{B}$
0–1	−32.6806	−0.0936	−0.0392	3.9347	−3.3688	5.7151
1–2	−50.3521	−0.2373	−0.1994	−2.8398	−4.7588	14.8659
2–3	−28.5767	−0.2283	−0.1016	−3.8268	−3.6163	19.4317
3–4	−25.7544	−0.0282	−0.0796	−4.5703	−2.2698	24.2711
4–5	−28.9198	−0.0371	−0.0725	−4.9651	0.7894	27.6308
5–6	−23.4521	−0.0310	−0.0356	−3.5320	0.1575	19.1554
6–7	−17.061	−0.0443	−0.0142	−2.6841	−0.7002	14.5734
7–8	−13.2486	−0.0015	−0.0130	−1.9060	−1.1391	12.8616
8–9	−11.6182	−0.0099	−0.0129	−1.6366	−2.0384	8.3779
9–10	−12.2738	−0.0020	−0.0122	−1.8312	−0.3261	8.8003

**Table 5:** Bathymetric capability and efficiency assessment of SBLS-1 at two test fields.

Test Fields	Trajectory Length (m)	Number of Echo Waveforms	Detection Ratio (%)	Laser Point Density (Points/m)
Weifang	3671.4	102,454	81%	22.6
Qingdao	1781.5	84,579	87%	41.2

## Data Availability

The data used in this study are available on request from the corresponding author.
